# Effects of HMGB1/TLR4 on secretion IL-10 and VEGF in human jaw bone-marrow mesenchymal stem cells

**DOI:** 10.1590/1678-7757-2023-0304

**Published:** 2024-01-29

**Authors:** Jingjing Kong, Wei Cheng, Lianzhen Chang, Jingyi Yu, Ronglin Wang, Jianli Xie

**Affiliations:** 1 Jinan Stomatological Hospital Department of Prosthodontics China Jinan Stomatological Hospital, Department of Prosthodontics, Shandong Province, China.; 2 Jinan Stomatological Hospital Department of Periodontist China Jinan Stomatological Hospital, Department of Periodontist, Shandong Province, China.

**Keywords:** HMGB1, JBMSCs, TLR4, IL-10, VEGF, Peri-implantitis

## Abstract

**Objective:**

We aimed to investigate the regulatory effects of HMGB1/TLR4 signaling pathway on the expression of IL-10 and VEGF in human bone marrow mesenchymal stem cells.

**Methodology:**

Human JBMSCs were isolated and cultured. Then, HMGB1 was added into the JBMSCs culture medium, and the protein and mRNA expression levels of IL-10 and VEGF were assessed. Moreover, cells were pretreated with a specific TLR4 inhibitor (TAK-242), and the expression changes of IL-10 and VEGF were compared.

**Results:**

Compared with the control group, exposure to HMGB1 in human JBMSCs up-regulated TLR4, IL-10, and VEGF secretion at both protein and mRNA levels (P<0. 05). In addition, the increased expression of IL-10 and VEGF could be restrained in TAK-242 group compared with the HMGB1 group (P<0.05).

**Conclusions:**

The results indicated that HMGB1 activate TLR4 signaling pathway in Human JBMSCs, which plays a regulatory role in cytokines expression.

## Introduction

The high mobility group (HMG) proteins are a superfamily of DNA-binding proteins in the cell nucleus, and these proteins promote nuclear transcription processes by interacting with DNA.^1,2^ High mobility group box1 (HMGB1) is an important member of HMG, being ubiquitous and highly conserved, in addition to presenting the capacity to be released to the extracellular environment by various cell types via two pathways, such as mononuclear cells, neuroblastoma cells, fibrosarcoma cells, osteoclasts, and osteoblasts.^3^ Studies have found that extracellular HMGB1 can be passively released by necrotic cells, and various cells secreted the protein actively after being stimulated by inflammation, stress, and injury.^4,5^ In addition, HMGB1 functions as a damage related molecular pattern (DAMP) protein and performs various cell compartment specific functions following the extracellular secretion. The research showed that HMGB1 can activate two cell surface receptors, the receptor for advanced glycation end products (RAGE), and the toll-like receptor 4 (TLR4).^6,7^ It leads to the secreting of a range of different mediators into the extracellular microenvironment, like chemokines, cytokines, and growth factors. These mediators, such as Interleukin 10 (IL-10) and Tumor necrosis factor α (TNF-α), consequently influence the intensity and duration of responses of mediating natural and adaptive immunity, damaging tissue repair, stem cell proliferation, and migration.^8,9^ Given all the evidence for the role of HMGB1 and its cell-stimulating properties in regulating immune and healing responses, the recognition of HMGB1 by receptors, followed by the secretion of downstream cytokines, appears to be a key step in triggering the inflammatory and healing processes of many diseases.

In the past few decades, the use of dental implants has become a common treatment for patients with complete edentulous and dentition defects. Ti-based dental implants have been widely used in clinical treatment with remarkable success due to their osseointegration capacity.^10^ However, oral implants have a failure rate, which is often caused by peri-implantitis, and the incidence of patients diagnosed with peri-implant diseases is increasing. Controlling inflammation and promoting bone tissue regeneration are urgent clinical issues. Jaw bone-marrow mesenchymal stem cells (JBMSCs) are adult stem cells that exist in the maxillary and mandibular bones, presenting multidirectional differentiation, immune regulation, and secretion of cytokines.^11,12^ Besides, JBMSCs also show convenient collection of materials, low immunogenicity, and broad application prospects in repairing the cranial and maxillofacial bone defect, regenerating periodontal tissue, and improving the success rate of oral implants, which have attracted widespread attention in basic and clinical studies.^13,14^ However, few studies on HMGB1 effects on the function of JBMSCs are available, especially on its secretion of cytokines.

A study shows that levels of IL-1β, IL-6, and TNF-α in subjects with peri-implantitis were significantly elevated compared with the healthy control group.^15^ Therefore, the expression of cytokines can also be measured to assess the degree of inflammation and recovery after treatment. IL-10 plays a role in the suppression of immune and anti-inflammatory reactions by inhibiting the activation of inflammatory cells, inhibiting the antigen presentation of mononuclear macrophages and the release of TNF-α, IL-1β, IL-6, IL-8, and other inflammatory mediators.^16,17^ Vascular endothelial growth factor (VEGF) promotes angiogenesis and plays a key role in maintaining vascular permeability and endothelial cell barrier, in addition to being involved in inflammatory response, wound healing, and tissue and organ growth and development.^18,19^ Thus, JBMSCs can play an anti-inflammatory role and promote the repairing of injured tissue by secreting these cytokines. Therefore, we designed this experiment to analyze the secretion of IL-10 and VEGF by JBMSCs. Initially, we explored the regulatory mechanism of HMGB1 on the secretion of cytokines, aiming to provide new ideas for the treatment of peri-implantitis.

## Methodology

### Isolation and culture of human jaw bone-marrow mesenchymal stem cells

The experiments were approved by the Research Ethics Committee of Jinan Stomatological Hospital (JNSKQ.2021-06-08). All patients were from Jinan Stomatological Hospital. All participants were informed about the study and signed informed consent forms. Mandibular bone fragments were collected from 15 patients (12-18 years old) with impacted wisdom tooth extraction who were required to remove bone resistance. Bone fragments were washed three times by phosphate-buffered saline (PBS). All alveolar bone fragments were placed on a mesh covered with 4 - (2-hydroxyethyl) - 1-piperazine ethanesulfonic acid (HEPS) medium, and JBMSCs were extruded from the bone fragments by centrifuging at 2500 rpm. Cells were inoculated on a 25 cm^2^ culture flask containing DMEM (Gibco, NY, USA) medium, then added with 15% heat inactivated fetal bovine serum (FBS, Gibco, New York, USA) and antibiotics (penicillin 0.1 g/L; streptomycin 0.1 g/L) at 37°C in the incubator. When cells reached confluence, they were considered passage zero. When the cell fusion reached more than 80%, cell passage was performed with trypsin. Cells from passages three to five were used for subsequent experiments.

### Identification of human JBMSCs

The third generation human JBMSCs were used for detection. Cells were digested and neutralized with trypsin, washed with PBS, and the supernatant was poured away. The cells were resuspended with PBS and cell density was adjusted to 1×10^6^ /mL. The cells were subpacked into a flow tube at 500 μL per tube, and the fluorescent direct label CD34, CD45, CD90, and CD105 antibodies were added to the flow tube at 5 μL, respectively, and incubated at room temperature for 30min in dark light. The cells were washed twice with PBS and then resuspended. Flow cytometry was used to detect stem cell-related surface markers.

### Real-time quantitative PCR

**Figure 1:**
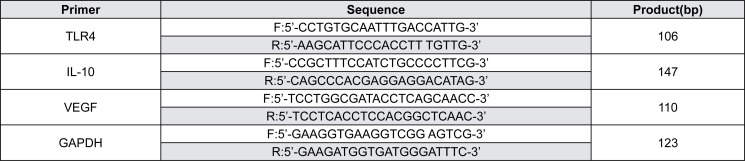
Primers used in this experiment

Human JBMSCs were seeded at a density of 1×10^5^ cells/cm^2^ in 24-well plates and incubated in DMEM with 10% FBS. Three groups were included in this study. In the control group, the cells were cultured in conventional medium for 48 h. In the HMGB1 group, the cells were incubated in the medium containing HMGB1 (Biovision, CA, USA,) for 48 h.^20^ In the TAK-242 group, the cells were pretreated with 1 μM TAK-242 (Invitrogen, San Diego, CA, USA), a specific inhibitor of TLR4, for 2 h before HMGB1 (200 ng/mL) stimulation for 48 h. The mRNA of TLR4, IL-10, and VEGF were measured. Following manufacturer's protocol, total RNA was extracted from cells of each group using Trizol (Invitrogen, Carlsbad, CA, USA). Then, the first complementary DNA strand (cDNA) was synthesized by using SYBR(R) Prime Script™ RT reagent Kit (Takara, Osaka, Japan). Quantitative real-time PCR using SYBR Green I dye (Takara, Osaka, Japan) was used to analyze the levels of target mRNA of TLR4, IL-10, and VEGF. Figure 1 presents the primer pairs used in the experiment.

### Western blot analysis

TLR4, IL-10, and VEGF were measured by Western-blot and compared to the amount of GAPDH, which was treated as protein loading control. Cells were collected and lysed with a lysis buffer. Then, the lysates were centrifuged for 30 min at 10,000 rpm. After, 5μg of protein was electrophoresed in 10% sodium dodecyl sulfate-polyacrylamidegel electrophoresis gel (SDS-PAGE), which was transferred onto a polyvinylidene difluoride membrane (Bio-Rad, Hercules, CA, U SA). The membrane was sealed in nonfat milk with Tris-buffered saline/0.05% Tween 20 (TBST), then incubated with primary antibodies to TLR4, IL-10, VEGF, and GAPDH (1:1000, Cell Signaling, MA, USA). These were incubated with secondary antibodies (1:5000, Santa Cruz, CA, USA). The protein fully reacted with the chemiluminescence (ECL) solution and was exposed to X-ray film. The relative protein levels were quantified with Image J software.

### Enzyme-Linked Immunosorbent Assays (ELISAs)

The IL-10 and VEGF secreted to cell culture medium were measured. Cell-free culture media from each sample were collected and stored at −80°C for testing. A commercial ELISA kit (Uscn, Hubei, China) was used to measure proteins expression levels following manufacturer's instructions.

### Statistical analysis

Each experiment was conducted at least three times, and the data were expressed using mean and standard deviation. Two-tailed Student's t-tests were used to compare normally distributed data. Data analysis was conducted in SPSS statistical software package version 18.0, and p<0.05 was considered significant.

## Results

### Morphological observation of human JBMSCs

**Figure 2:**
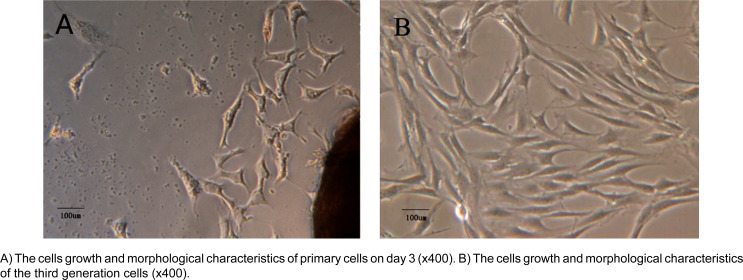
The morphology of human JBMSCs

Figure 2 shows the cell growth. After three days, when the medium was changed for the first time, crawling behavior was noted in fibrillar cells when observed under a microscope (Figure 2 A). After five days, the adherent monolayer cells were observed under microscope, and the fusiform JBMSCs seemingly fused themselves at the edges. After subculture, the cells proliferated in a vortex aggregation pattern (Figure 2 B).

### Identification of human JBMSCs

**Figure 3:**
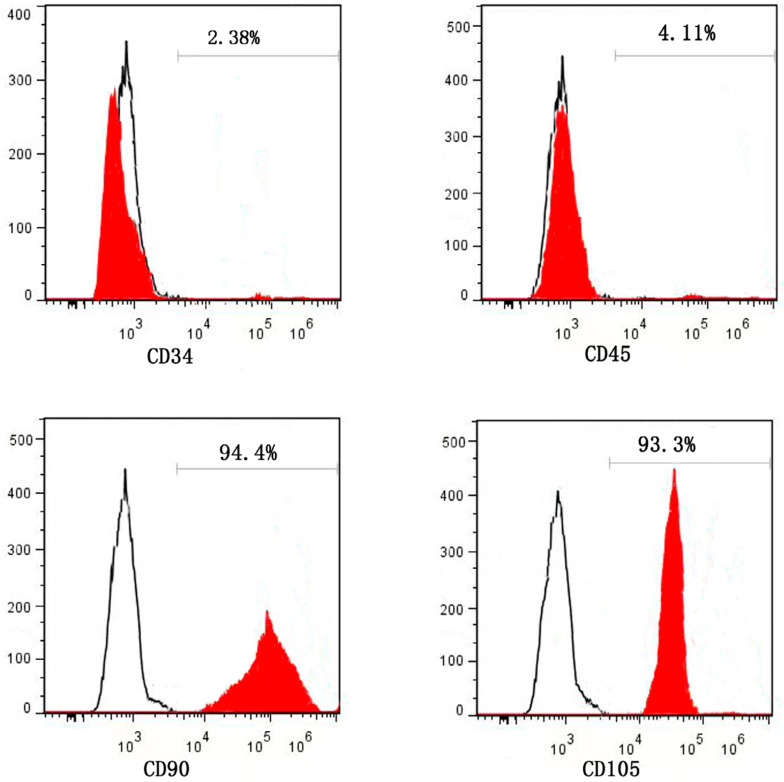
The results of cell surface markers were detected by flow cytometry

The surface markers of hematopoietic stem cells (HSCs) include CD34 and CD45, and the surface markers of mesenchymal stem cells (MSCs) include CD90 and CD105. Human JBMSCs surface markers were detected by flow cytometry. The results showed a high expression of CD90 and CD105, and positive expression rates of 94.4% and 93.3%, respectively. However, CD34 and CD45 showed low expression, with positive expression rates of 2.38% and 4.11% (Figure 3), respectively. This indicated that the cells obtained in the experiment were consistent with the characteristics of BMSCs and could be used in subsequent experiment.

### HMGB1 stimulates TLR4 expression in human JBMSCs

**Figure 4:**
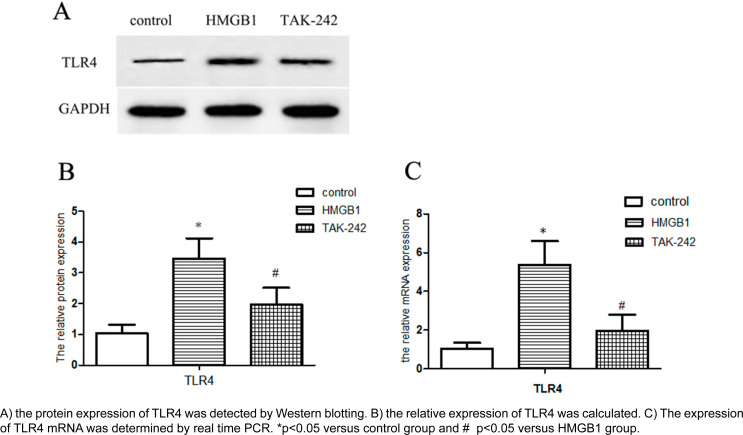
TLR4 Expression in human JBMSCs with HMGB1 stimulation

In this study, JBMSCs were stimulated with HMGB1 in a concentration of 200 ng/mL for 48 h. The results show that exposure to HMGB1 upregulated TLR4 secretion, at both protein and mRNA levels, compared with the control group (p<0.05). In the TAK-242 group, pretreating cells with TAK-242 reduced the protein expression of TLR4 compared with the HMGB1 group. The mRNA expression of TLR4 was also reduced, consistent with the protein change. Figure 4 shows the results.

### Effect of TLR4 on the expression of IL-10 and VEGF in human JBMSCs.

**Figure 5:**
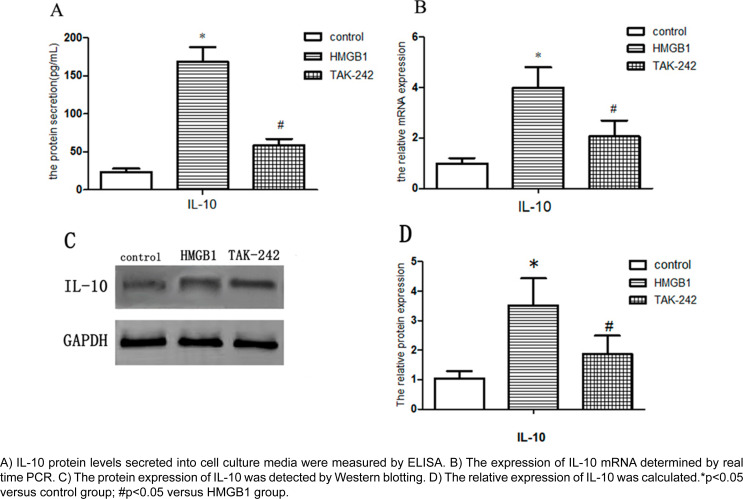
Effect of TLR4 on the expression of IL-10

**Figure 6:**
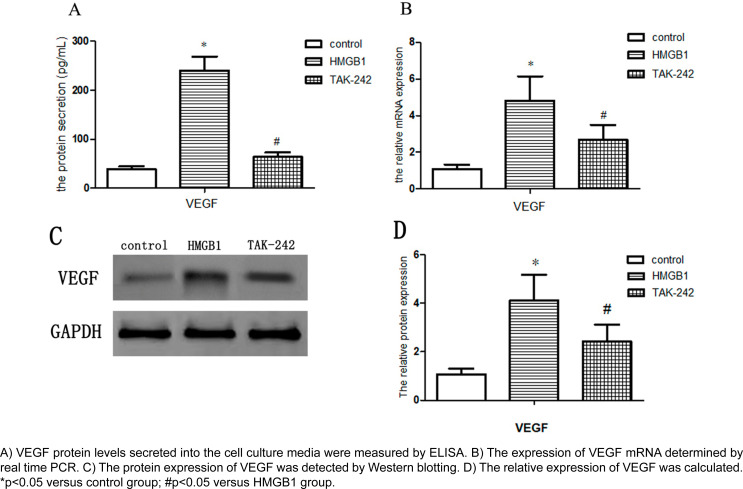
Effect of TLR4 on the expression of VEGF

The total quantities of IL-10 and VEGF secreted protein in cell culture medium was measured by ELISA and Western Blot. As shown in Figure 5 A, C, and D, the expression of IL-10 in the HMGB1 group improved. However, compared with the HMGB1 group, the expression of IL-10 was decreased in the TAK-242 group. The same result was also found in the mRNA expression of IL-10 (Figure 5 B). To further investigate the roles of TLR4 in the expression change of VEGF, the protein and mRNA levels were measured by ELISA, Western blot, and Real-Time PCR (Figure 6). Consistent with the IL-10 secretion, the protein and mRNA expression of VEGF showed the same trends. These findings suggest that the upregulated TLR4 can lead to a substantial increase in IL-10 and VEGF secretion, and these cytokines expression could be governed by TLR4 signaling triggered by HMGB1.

## Discussion

Research showed that JBMSCs hold stronger self-renewal and osteogenic capacities compared with bone marrow mesenchymal stem cells (BMSCs) derived from long bone.^21^ In a study conducted by Du, JBMSCs showed stronger angiogenesis capacity than BMSCs derived from ilium bone *in vitro* culture and *in vivo* microenvironment.^22^ Moreover, JBMSCs can also affect the surrounding microenvironment by paracrine secretion of cytokines and exosomes, thus affecting various physiological activities of cells. Exosomes derived from JBMSCs can play a role in the cell communication between JBMSCs and iliac BMSCs, enhance the osteoblastic effect of iliac BMSC, promote the expression of alkaline phosphatase (ALP) and osteoblastic genes, and promote the formation of new bone *in vivo* after transplantation.^23^ Given the roles of IL-10 and VEGF in anti-inflammatory and vascular regeneration processes, we examined the expression changes of these two cytokines by JBMSCs. The results suggested that JBMSCs have the capacity to secrete cytokines such as IL-10 and VEGF into the surrounding environment in the presence of external stimulation. Therefore, further research on the molecular mechanism behind JBMSCs' secretory function is needed to enhance their secretory effect, improving their repairing effects on damaged tissues.

HMGB1 and its downstream signaling pathways play an important role in the occurrence and development of many illnesses. It is proposed that HMGB1 shows dual effects on osteoclasts, directly inducing differentiation by recognizing TLR4 and RAGE, and enhancing pro-osteoclastogenic cytokine secretion of osteocytes indirectly.^24^ In TLR4-positive cancer cells, HMGB1 induced the expression of galectin-9 to help cells escape immune attacks, whereas, in malignant cells lacking TLR4, HMGB1 activated TLR4-expressing myeloid cells present in the tumor microenvironment indirectly, leading to the same effect.^25^ Elevated HMGB1 and RAGE levels have been demonstrated in plasma of acute myocarditis patients and mouse model of myocarditis; furthermore, ablation of RAGE nearly abolished Tnl-induced myocardial inflammation and consecutive heart failure.^26^ Researchers designed *in vitro* studies in rat models of transplant arteriosclerosis, finding that HMGB1 stimulated MSCs to migrate and differentiate endothelial cells via RAGE signaling, which was beneficial for successful application in cell therapy for transplant arteriosclerosis.^20^ Fujita, et al.^27^ (2015) has demonstrated that HMGB1 can recruit MSCs expressing platelet derived growth factor receptor-α on the cell membrane surface to the site of inflammation, stimulate MSCs to secrete TSG6, inhibit tissue inflammation, and enhance the immunosuppressive effect of MSCs. Based on the above research analysis, we found that the effects of HMGB1 on the body environment are complex and have different effects on different effector cells. Therefore, we explored the effect of HMGB1 on human JBMSCs by *in vitro* experiments. The results reflected that the mRNA and protein expression level of IL-10 and VEGF is significantly increased.

To verify whether TLR4 triggered signal transduction can upregulate the expression of IL-10 and VEGF, we pretreated JBMSCs with a specific inhibitor to TLR4 (TAK-242) before adding HMGB1 to cells. The results have shown that TAK-242 can inhibit HMGB1-enhanced IL-10 and VEGF expression significantly at both mRNA and protein levels. These results may represent an important information that TLR4 is a receptor that is responsible for the pathways of signaling transduction intracellularly to regulate IL-10 and VEGF expression. However, little is known about whether the TLR4 can regulate cytokines expression in the experimental animal model with peri-implantitis.

Peri-implantitis is a mixed infectious disease that causes inflammatory process in both soft and hard tissues around a dental implant, resulting in some degree of bone loss. Risk factors include dental plaque, history of periodontitis, smoking, and diabetes, etc. Peri-implantitis is characterized by redness, bleeding on gentle probing, with or without suppuration, a peri-implant pocket depth greater than or equal to 5 mm, as well as X-ray evidence of marginal bone loss greater than or equal to 2 mm.^28^ The results of a meta-analysis systematic review showed that the weighted average prevalence of peri-implant mucositis and peri-implantitis was 43% (range: 19-65%) and 22% (range: 1-47%), respectively.^29^ In addition, in a series of 86 patients with long-term follow-up (i.e., 21-26 years), the prevalence of peri-implant mucositis and peri-implant inflammation was 54.7% and 22.1%, respectively.^30^ The severity of bone loss caused by peri-implant inflammation increased with the duration of the disease. Peri-implantitis treatment comprises many methods, including mechanical debridement, combined treatment of mechanical debridement and drugs, as well as surgical treatments such as resection and regenerative surgery.^31–33^ In recent years, the therapeutic value of stem cells has attracted the attention of many scholars. In this study, JMBSCs were isolated and cultured *in vitro*, and the regulation of cytokine secretion was studied. The results indicated that HMGB1 activates TLR4 signaling pathway, which plays a regulatory role in the expression of IL-10 and VEGF. We will refine the relevant experimental data in the following research. For example, JMBSCs will be co-cultured with osteoblasts or gingival fibroblasts to observe the effect of cytokines secreted by JMBSCs on surrounding tissue cells. We will establish an animal model of peri-implantitis with isolate and culture JMBSCs and implant them into the site of peri-implant inflammation. Alternatively, we can use HMGB1 to pre-treat JMBSCs and transplant them and test their capacity to repair damaged tissues. We hope to enhance the secretion effect of JMBSCs and up-regulate the expression level of various cytokines by specific amounts to further play a beneficial role in regulating inflammatory factors and promoting angiogenesis in the process of repairing damaged tissues. We hope these results will provide new ideas for the treatment of peri-implantitis.

## Data Availability

All data generated and analyzed during this study are included in this published article.
